# Effect of heavy-ion beam irradiation on the level of serum soluble interleukin-2 receptors in hamster cheek pouch carcinoma model

**DOI:** 10.3892/br.2014.238

**Published:** 2014-02-17

**Authors:** XIAOLI AN, MINGXIN LI, NA LI, BIN LIU, HONG ZHANG, JIZENG WANG

**Affiliations:** 1 College of Stomatology, Lanzhou University, Lanzhou, Gansu 730000, P.R. China; 2 Institute of Modern Physics, Chinese Academy of Science, Lanzhou, Gansu 730050, P.R. China; 3 Institute of Solid Mechanics, School of Civil Engineering and Mechanics, Lanzhou University, Lanzhou, Gansu 730000, P.R. China

**Keywords:** soluble interleukin-2 receptors, cheek pouch carcinoma, heavy ion irradiation, immune status

## Abstract

Soluble interleukin-2 receptor (sIL-2R) is a glycoprotein derived from α chain of interleukin 2 receptors of mononuclear as well as T-cell membranes. The aims of this study were to detect the changes of serum soluble interleukin-2 receptor (sIL-2R) levels following heavy-ion beam irradiation in the hamster model with cheek pouch carcinoma, as well as to examine the impact of immune status of the hamster cheek pouch carcinoma model using heavy-ion beam irradiation. sIL-2R serum levels were detected by radioimmunoassay (RIA) in 40 hamsters bearing cheek pouch carcinoma prior to and following exposure to heavy-ion beam irradiation, and 8 normal animals served as the control. The sIL-2R serum level in hamster cheek pouch carcinoma model was significantly increased as compared to the normal control group (P<0.05). Results showed that an increase in the irradiation dose led to a gradual decrease in the sIL-2R serum level. Additionally, a statistical significance was observed compared to the tumor group (P<0.05). In conclusion, alterations in serum sIL-2R expression have an effect on the hamsters cheek pouch carcinoma model subsequent to heavy-ion beam irradiation. An increase in the irradiation dose indicated a decreased tendency in serum sIL-2R content. Detection of serum level changes may lead to an improved understanding of heavy-ion irradiation *in vivo* immune status, which is crucial for clinical diagnosis and prognosis. It can also provide a sensitive indicator to help estimate the effects of heavy-ion cancer targets.

## Introduction

Soluble interleukin-2 receptor (sIL-2R) is a glycoprotein which derived from α chain of interleukin 2 receptors of the mononuclear cell membranes as well as the T-cell membranes and the molecular weight is 45 kDa (?). sIL-2R is crucial in lymphokines in the lymphocyte culture fluid and blood circulation, and plays a significant role in regulating cell immune function. The secretion of sIL-2R increased when lymphocytes stimulated by a specific antigen or mitogen. Regulation of the expression of the sIL-2R in IL/sIL-2R system is crucial and is generally considered as another important indicator involved in the activation of the immune system (1,2). Imbalance of the IL-2/sIL-2R system is capable of inducing abnormalities of the immune function of tumor cells in the body, resulting in the immune escape of tumors. Previous studies showed that the expression of sIL-2R levels was altered in the serum of malignant solid tumors, autoimmune diseases and skin melanoma patients and was associated with the immune state of the body (3–6). This study aimed to determine the sIL-2 serum level in 40 hamster cheek pouch carcinoma models and investigated the effect of heavy-ion beam irradiated tumor-bearing golden hamster immune status.

## Materials and methods

### Reagents

Reagents and equipment used in the present study included: 7,12-dimethyl-1,2-benzanthracene (DMBA) 090 (Sigma, St. Louis, MO, USA), acetone of analytical grade (Columbine Canton of Bose National Instrument Co., Ltd., ?), ELISA kit (Wuhan Boster Biological Technology Ltd., Wuhan, China), lotion self with 0.01 M PBS (1,000ml H_2_O followed by Tris 1.2 g NaCl 8.5 g, with the pH adjusted to 7.2–7.6 and concentrated hydrochloric acid, ~700 μl), EL×800 microplate reader (BioTek Instruments Inc., Winooski, VT, USA), EL×50 microplate strip washer (?), G5-2A low-speed centrifuge (Beijing Medical Centrifuge Factory), 200 μl pipettes NPX-200 (Nichiryo Co., Ltd., Koshigaya, Japan), 75% ethanol (Tianjin Hong Yan Chemical Reagent Factory, Dongli, China), 1 ml of medical syringe (Changzhou Chunguang Medical Equipment Co., Ltd., Changzhou, China), as well as calipers, dividers and ruler (The Beijing Jingxicheng Optical Instrument Co., Ltd., Beijing, China).

### Experimental animals

#### Selection and grouping of the animals

The study included 48 (n=24 males and females, respectively, per group) normal 6- to 8-week-old Syrian golden hamsters (purchased from the Lanzhou Institute of Biological Products, Lanzhou, Gansu, China), with a weight of 80–100 g. The animals were raised at a room temperature of 24±2°C, under 12-h light and dark cycle conditions. The hamsters were randomly divided into five groups prior to irradiation, at the proposed irradiation doses of 0, 4, 6, 8 and 12 Gy.

#### Establishment of the experimental animal model

DMBA acetone (0.5%) was prepared according to a previously published method (7). The prepared DMBA acetone liquid was coated on the right side of the golden hamster cheek pouch mucosa with a 4 Brush Pen, three times a week and consecutively for 16 weeks in order to establish the tumor model. Right cheek pouch tissue was removed prior to the experiment and analyzed using hematoxylin and eosin staining as per the histological grade determination criteria [according to the WHO (1996) of oral precancerous lesions and the Banoczy method] (8,9).

#### Heavy-ion irradiation

Heavy ion was irradiated on a biological irradiation terminal device located at the heavy-ion irradiation facility laboratory (HIRFL) of the Lanzhou Institute of Modern Physics Laboratory, China. A ^12^C^6+^ ion beam was produced and used for animal irradiation. The energy generated was 235 MeV, linear transfer energy (LET) was 155 keV/μm and the absorbed dose rate was 3 Gy/min respectively. The dose was monitored using an air ionization chamber.

### Methods

#### Specimen collection

In the first four weeks following heavy-ion irradiation all 48 animals were fasted. Venous blood (2 ml), without anticoagulant, was drawn and incubated overnight at 4°C, then centrifuged for 10 min at 1,000 rpm. Serum was then collected and stored at −20°C.

#### Detection methods

Serum sIL-2R was measured by radioimmunoassay (RIA) using a radioimmunoassay kit (Beijing Huaying Biotechnology Research Institute, Peking, China); GC-911 gamma counter (HKUST Innovation Co., Ltd., Zhongjia, China); and KDC-2044 low-speed refrigerated centrifuge (HKUST Innovation Co., Ltd.). Measurements were monitored by quality control.

#### Statistical analysis

SPSS 15.0 statistics was used for data analysis. Data were expressed as mean ± standard deviation (SD). Statistical analysis was performed using the Student’s t-test and variance analysis. Spearman correlation analysis was used to determine the correlation between the groups at a signficance level of α=0.05.

## Results

### Expression levels of serum sIL-2R in the tumor and control groups

The results showed that sIL-2R serum levels of the simple tumor-bearing and blank control groups were: (343.84±34.58) and (124.26±10.41) μ/ml respectively. sIL-2R levels of the simple tumor-bearing group were significantly higher than those of the blank control group (P<0.01) (Table I).

### Serum sIL-2R expression between the tumor and irradiation groups

The sIL-2R serum levels of the simple tumor-bearing group (0 Gy) and irradiation group (4, 6, 8 and 12 Gy) were: (343.84±34.58), (270.32±20.27), (212.10±25.68), (198.83±13.64) and (167.43±11.50) μ/ml respectively. The results show a decrease in the sIL-2R serum level with the increase of the irradiation dose between the two groups. Statistically significant differences were observed when compared with the blank control group (P<0.05) (Table I).

### Association of serum sIL-2R level changes in the different irradiation dose groups

**Figure 1:**
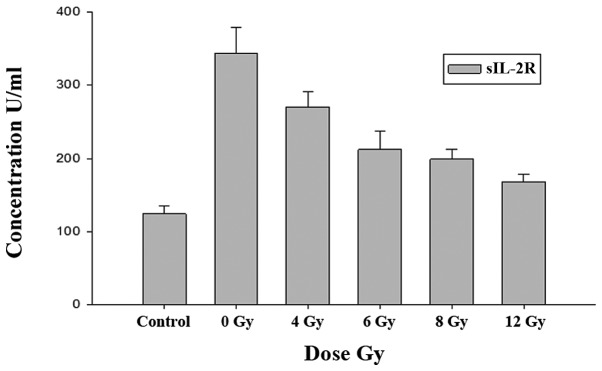
Association of serum soluble interleukin-2 receptor (sIL-2R) level changes in the irradiation dose groups. Significant differences between the simple tumor-bearing group (0 Gy) and the control group (P<0.01); compared with 4, 6, 8 and 12 Gy (P<0.05), with statistical significance.

An increase in the irradiation dose resulted in a gradual decrease in sIL-2R serum levels in the irradiation groups. Expression levels of sIL-2R and the irradiation dose were positively correlated (rs=−0.949, P=0.001<0.01), as analyzed by the Spearman correlation analysis. Statistically significant differences were observed between the simple tumor-bearing and blank control groups (Fig. 1).

## Discussion

The occurrence and development of tumors are closely associated with immune function and patients with malignant tumors experience immune dysfunction. Tumor cells secrete various immunosuppressive factors by themselves or are induced by these factors, resulting in a decrease in the immunogenicity of the tumor cells and leading to immune escape of the tumors. sIL-2R is a glycoprotein shedding from membrane interleukin-2 receptor (mIL-2R) which is on the the surface of cytomembrane together with the enzyme activity. Only a small number of sIL-2R were shed from the surface of activated normal lymphocytes, most of which were from malignant tumor cells, and circulated in the blood. Therefore, sIL-2R is regarded as an active biological response marker of the host and malignant tumours and is one of the significant signals in the activated immune system of the organism.

Recent studies (?) have shown that sIL-2R has a bidirectional immunoregulatory action, and competes with IL-2R when combined with IL-2, reducing the blood concentration of IL-2 and lymphocyte activation, while releasing IL-2 when IL-2 is at a low level, thereby achieving a self-protective function in order to maintain the balance of the immune response. It is believed (10,11) that sIL-2R competes with mIL-2R when combined with IL-2, similar to blocking agents, thereby neutralizing IL-2 activated T cells and regulating the immune response of the IL-2 antagonist effect, and weakening the organism’s autocrine effect. Furthermore, the production of sIL-2R involves shedding of the membrane receptor mIL-2R p55 chain. An increase of sIL-2R accelerates the expurgation of sIL-2 and then restrains the activation and multiplication of T cells. sIL-2R shedding from membranes resulted in activated lymphocytes recovering to a static condition or lymphocyte exhaustion, thereby reducing the immunocompetence of T cells and leading to abnormal immune function.

The cell immune response has an inhibitory role in malignant tumors. Immune cells lose the ability to regulate the expression of IL-2R chain, resulting in abnormal immune cells being highly expressed in IL-2R chain and released into the bloodstream, resulting in high levels of serum sIL-2R. sIL-2R levels are usually positively correlated with the progress of clinical stages and prognosis, although the mechanism by which this occurs is not clear. Tumors are known to activate the immune cells, such as T cells, *in vivo* and release excessive amounts of sIL-2R, resulting in a high expression of sIL-2R. sIL-2R levels decreased when the tumors disappeared, although its content increased when tumor relapse occurred. In their study, Li Ling *et al* (13) demonstrated that high sIL-2R serum levels were significantly decreased following removal of the tumors. Tartour *et al* (14), Murakami *et al* (15) and Grotowski and Piechota (16) reported that the sIL-2R serum levels of head and neck, gastric and colorectal cancer patients were significantly higher compared to the control groups and showed an increase with progression of clinical stage. Lai *et al* (17) revealed that sIL-2R serum levels in nasopharyngeal carcinoma patients were significantly higher compared to the normal controls and was significantly increased with progression of clinical stage, with stage III-IV>I-II sIL-2R serum levels being lower subsequent to radiotherapy. In the present study, results showed that an increase in the irradiation dose following heavy-ion beam irradiation resulted in a decrease in the sIL-2R serum levels of the tumor-bearing golden hamsters. Our findings are in concordance with those of Xu Mei *et al* (18) who reported changes in sIL-2R serum levels in esophageal cancer patients prior to and following radiotherapy.

We analyzed the related factors and possible reasons for heavy-ion beam irradiation affecting changes in sIL-2R levels (1). sIL-2R is involved in the regulation of the body’s immune status as when it is significantly expressed in tumors it is able to reverse the activated state of immune cells to a dormant one. As heavy-ion beam irradiation removed the tumor burden of tumor-bearing golden hamsters, the secretion of sIL-2R was reduced by blocking tumors to the activation of T lymphocytes; however, the content of IL-2 increased. Heavy-ion beam irradiation resulted in the tumor losing its ability to remove immune suppression more than direct immunosuppressive effects and was beneficial for immune regulation. Heavy-ion radiation damaged the DNA of the target cells, leading to cell mutation or death (19,20), and triggered the body to make feedback regulation for this signal and the result was activated, initiating the IL-R/IL-2R system, make sIL-2R combine with IL-2 increased, to reduce the content of serous sIL-2R and activation effect of T lymphocyte. Together with the disappearance of the tumor burden, autocrine effects in the body weakened, and the sIL-2R serum levels decreased. This suggested that irradiation potentially activates and starts the body stress repair system and enhances the immune effect.

sIL-2R is a crucial immunologically reactive substance, which together with other factors including mutual regulation and mutual restraint, forms a complex signaling pathway network. Heavy-ion irradiation generated significant radiation damage effects on tumors, alterated tumor microenvironment and influenced metabolites, thereby agitating immune cells to the expression of the IL-2R chain. The content of the expression appeared abnormal, as well as the progress of the clinical stage, degree of immune inhibition and the body’s own secretion amount of IL-2; irradiation dose, sensitivity of the body to the irradiated and irradiated site be related. sIL-2R is an indicator for the assessment of immune status of the tumor body cells and that real-time monitoring of sIL-2R levels may provide an understanding of the development of tumors and the immune status of the body. Thus, sIL-2R can also be used as an objective indicator to evaluate the efficacy of heavy-ion beam as cancer treatment and its effects on clinical prognosis.

## Figures and Tables

**Table I:** Comparison of serum soluble interleukin-2 receptor (sIL-2R) levels in the three groups (mean ± SD) (U/ml).

Group	No.	Dose (Gy)	sIL-2R
Blank control	8	-	124.26±10.41
Simple tumor-bearing	8	0	343.84±34.58^a^
	8	4	270.32±20.27
Tumor-bearing and irradiation	8	6	212.10±25.68^b^
	8	8	198.83±13.64^b^
	8	12	167.43±11.50^b^

a P<0.01, compared with the tumor group;

b P<0.05, compared with the blank control group.
